# Longer wings for faster springs – wing length relates to spring phenology in a long‐distance migrant across its range

**DOI:** 10.1002/ece3.1862

**Published:** 2015-12-08

**Authors:** Steffen Hahn, Fränzi Korner‐Nievergelt, Tamara Emmenegger, Valentin Amrhein, Tibor Csörgő, Arzu Gursoy, Mihaela Ilieva, Pavel Kverek, Javier Pérez‐Tris, Simone Pirrello, Pavel Zehtindjiev, Volker Salewski

**Affiliations:** ^1^ Department of Bird MigrationSwiss Ornithological InstituteSempachSwitzerland; ^2^ Zoological InstituteUniversity of BaselBaselSwitzerland; ^3^ Department of Anatomy, Cell & Developmental BiologyEötvös Loránd UniversityBudapestHungary; ^4^ Department of BiologyOndokuz Mayis UniversitySamsunTurkey; ^5^ Institute of Biodiversity & Ecosystem ResearchBulgarian Academy of SciencesSofiaBulgaria; ^6^ Vilová 246KněžmostCzech Republic; ^7^ Department of Zoology and Physical AnthropologyComplutense University of MadridMadridSpain; ^8^ SEO‐Monticola Ringing GroupAutonomous University of MadridMadridSpain; ^9^ Institute for Environmental Protection and Research (ISPRA)Ozzano dell'EmiliaItaly; ^10^ Michael‐Otto‐Institut in NABUBergenhusenGermany

**Keywords:** Aerodynamics, body size, ecomorphology, flight, *Luscinia megarhynchos*, timing

## Abstract

In migratory birds, morphological adaptations for efficient migratory flight often oppose morphological adaptations for efficient behavior during resident periods. This includes adaptations in wing shape for either flying long distances or foraging in the vegetation and in climate‐driven variation of body size. In addition, the timing of migratory flights and particularly the timely arrival at local breeding sites is crucial because fitness prospects depend on site‐specific phenology. Thus, adaptations for efficient long‐distance flights might be also related to conditions at destination areas. For an obligatory long‐distance migrant, the common nightingale, we verified that wing length as the aerodynamically important trait, but not structural body size increased from the western to the eastern parts of the species range. In contrast with expectation from aerodynamic theory, however, wing length did not increase with increasing migration distances. Instead, wing length was associated with the phenology at breeding destinations, namely the speed of local spring green‐up. We argue that longer wings are beneficial for adjusting migration speed to local conditions for birds breeding in habitats with fast spring green‐up and thus short optimal arrival periods. We suggest that the speed of spring green‐up at breeding sites is a fundamental variable determining the timing of migration that fine tune phenotypes in migrants across their range.

## Introduction

Ecological morphology and life history are main aspects in migration ecology dealing with different but interacting traits. Herein, ecomorphology focuses on the interrelationship of morphological variation among individuals, populations and species and the corresponding variation in their ecology (Leisler and Winkler 1985, 2003). A prominent example for this is the pointedness of wings of birds that corresponds to their migratory behavior (e.g., Kipp 1959; Fiedler 2005; Förschler and Bairlein 2011). The life‐history perspective though focuses on the adaptive value of individual behavior, especially in timing of migration (Smith and Moore 2005). Morphology and life history are important parts within the optimal migration theory (Alerstam 2011), but surprisingly few studies tried to integrate both (e.g., Stolt and Fransson 1995; Matyjasiak et al. 2013).

Aerodynamic theory predicts that longer, pointed wings are more efficient for long flights than shorter, rounded wings (Norberg 1995; Pennycuick 2008). Therefore, longer wings are more important when travelling large distances within the annual cycle. Consequently, migrating species have longer wings compared to closely related but more sedentary species (e.g., Förschler and Bairlein 2011). The same pattern occurs on subspecies (Pérez‐Tris and Tellería 2001; Fiedler 2005) and population levels (Bowlin and Wikelski 2008), supporting the prediction that morphological traits enabling efficient (migratory) flight are under natural selection (Hedenström 2002).

The timing of arrival at and departure from various sites is important for migrants and particularly applies to breeding areas to match requirements with local food availability (Drent 2006; Bridge et al. 2010), to gain high quality territories or mates (Kokko 1999). Timing of annual cycle is crucial as almost all sites are seasonal to some degree and site specific phenology can vary greatly (Menzel et al. 2005). However, adjusting the timing of subsequent migratory steps and the arrival at final destination is often challenging due to limited predictability of conditions at sites ahead (Kölzsch et al. 2015).

**Figure 1:**
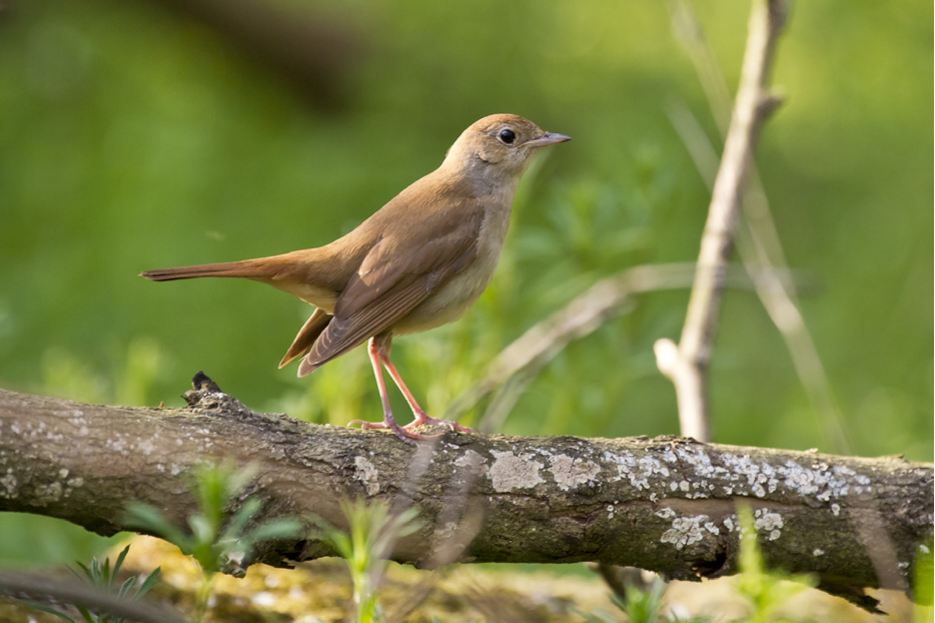
The common nightingale *Luscinia m. megarhynchos* is a Palearctic‐African migrant with a wide breeding range from western Europe to western Asia. (picture: Marcel Burkhardt, ornifoto.ch).

Morphological adaptations to migration have often been viewed under the perspective of optimal aerodynamic performance for efficient flight (Norberg 1995; Pennycuick 2008), for example to cover certain distances (e.g., Leisler and Winkler 2003). However, adaptations in phenotype according to seasonal environmental cycles and to life history features are rarely studied simultaneously. Here we combine phenology at breeding sites, migration distances and the need for optimal timing to explain the variation in morphology of a long‐distance migrant across its range. To this end, we explain wing morphology of nominate Common Nightingales (*Luscinia megarhynchos megarhynchos*) with the spring phenology of vegetation at breeding grounds from various sites along a gradient from oceanic to continental climate within Europe. The nightingale is a Palaearctic woodland species (Fig. 1) with three subspecies in Eurasia (Dickinson and Christidis 2014): the western (European) *megarhynchos* nominate subspecies is the smallest and the eastern Asian *golzii (*former *hafizi)* subspecies the largest (Loskot 1981). Nightingales spend the nonbreeding season in sub‐Saharan Africa in moist savannahs and savannah‐forest mosaic mainly south of 10°N (Walther et al. 2010). Ring recoveries and geolocation verified that populations breeding in western, southern‐central and eastern Europe use different flyways (Korner‐Nievergelt et al. 2012; Hahn et al. 2014). The longitudinal nonbreeding distribution mirrors the breeding distribution with western populations found in western Africa and eastern population in central Africa (Hahn et al. 2013).

We expect a continental gradient in wing length and body size from western to the eastern populations (Stresemann 1920; Eck 1975). However, longitude as such has no ecological relevance. Therefore, we aim to substitute longitude with variables of direct biological relevance which we relate to wing length variation. First, based on aerodynamic theory, we expect longer wings in populations with longer migration distances (Pérez‐Tris and Tellería 2001; Fiedler 2005). Migration theory predicts optimal arrival at final destination (e.g., Alerstam 2011) and, accordingly nightingales arrived at breeding sites to match the local spring phenology (Emmenegger et al. 2014). Thus, we secondly expect that spring phenology of the vegetation and the temporal food availability at breeding areas can co‐explain variation in wing length at continental scale.

## Materials and Methods

### Morphometric data

We analysed wing length as a flight related trait and tarsus length as representative of structural body size (e.g., Rising and Somers 1989; Freeman and Jackson 1990; Senar and Pascual 1997) of the nominate common nightingale *Luscinia m. megarhynchos* from 28 breeding sites across its entire range from Portugal (8.3°W) to central Turkey (36.0°E) and from southern Spain (36.5°N) to north‐western Poland (52.8°N). The field studies had been carried out before 1950′s (three studies), between 1950 and 2000 (12 studies) and after 2000 (13 studies, for sources, sites und numbers see Table S1).

We only considered measurements of adult birds (birds in the second year and older) collected between the second half of April and July to exclude adult on passage and juveniles. Wing length increases from first‐year to older birds in nightingales by on average, 0.11 mm between second‐year and older than second year birds (Dorsch 2010). As we lack information on age structure of adult breeders from all sites, we assume a similar proportion of second‐year and older birds on each site. Sex of captured nightingales was usually identified by the presence/absence of incubation patches, cloacal protuberances and singing after release.

The final data set comprised population means (± SD) of wing length (in parentheses: for tarsus length) of males from 25 (12) sites, of females from 18 (8) sites and of individuals with unknown sex from 4 (2) sites; originating from living birds (*n* = 21 sites) and from museum specimens (*n* = 7 sites). The museums specimens had on average 0.79 mm shorter wings than live birds likely caused by desiccation of the integument (95% CrI: −0.04 to 1.67, see Results). Although the difference between live and museum birds was marginally nonsignificant, we categorized data according to state (alive vs. museum) for subsequent statistical analysis. Tarsus length did not differ between living and museum specimen (average difference 0.07 mm, CrI: −0.40 to 0.51).

We modeled the geographic pattern of wing length across the breeding distribution of nightingales using the results from regression analysis (see below) for the status “living birds” and the species actual distribution maps provided by IUCN (2012).

#### Testing for allometry at the population level

Morphometric variables often show an allometric relation, that is, the proportion between the focal traits changes with size (due to physical constraints). We assessed the extent and direction of allometry between tarsus and wing length for males and females within six populations (Spain, France, Italy, Czech Republic, and two populations in Bulgaria). For four (males) and two (females) populations we found statistically significant (major axis) regressions with slopes being smaller than 1 (for males: 0.41 ± 0.19 [SE], for females: 0.28 ± 0.12 [SE]). Hence, the allometry effect was either absent or small, with birds that had longer tarsi having proportionally smaller wings.

### Minimum migration distance

The nonbreeding sites in sub‐Saharan Africa are unknown for most nightingale populations. Ring recoveries (Korner‐Nievergelt et al. 2012) and geolocation (Hahn et al. 2014) indicated the East‐West distribution during the nonbreeding season with partially overlapping ranges of neighboring populations broadly resemble the longitudinal distribution during breeding. We defined the minimum migration distance (Dist) in spring as the loxodromic distance between the northernmost nonbreeding range and the respective breeding area. As the northernmost nonbreeding range we selected the northern edge of cultivated land/cropland habitat from land cover maps (GlobCover project of ESA, http://due.esrin.esa.int/page_globcover.php). The minimum migration distance for each population was calculated as the mode of the 50 km bin frequency distribution of loxodromic distance between the northernmost non‐breeding area in Africa and the breeding site in Europe.

### Time and speed of local spring green‐up

Nightingales are insectivorous ground feeders and prefer deciduous woodlands and sometimes scrubland as breeding habitats (Cramp 1988). As nightingales commonly arrive after bud burst (V. Amrhein, unpubl. data), habitat suitability in spring is likely related to the development of vegetation, for both food availability and predator protection. Thus, the spring phenology of primary production, that is the increase in plant productivity should indicate an increase in breeding habitat suitability. We used the date and the speed of local spring green‐up to characterize breeding habitat phenology. We extracted Normalized Differenced Vegetation Index (NDVI) data between 1982 and 1992 from the GIMMS dataset (Tucker et al. 2004); the time period encompassed the average period of the morphometric studies. Subsequently, we calculated 11‐years NDVI averages for each site and week to obtain the general phenology of each site. We defined the local spring green‐up (GU_time_) as the time of the steepest increase in primary production determined by fitting a logistic regression to NDVI data over time. Additionally, we used the slope of this logistic regression to quantify the speed of spring green‐up (GU_speed_) (Pettorelli et al. 2005), that is a shallow slope indicating a slow greening of the vegetation.

### Local insect phenology

Ambient temperatures trigger the activity of insect imago as well as the development of their larvae and thus can serve as a proxy for the appearance of food for insectivores. For each site, we determined the time when insects become available for insectivores as the day of the year (FA_first_ – the first food available) when the mean daily air temperature first exceeds 10.4°C, the lower developmental threshold temperature of insects (Jarošík et al. 2011). Additionally, we defined the onset of high availability of insects (FA_high_) as the time when 59.1 degree‐days above this developmental threshold temperature were accumulated, which corresponds to the average thermal requirement for hatching of first stages of insect larvae (Jarošík et al. 2011). To this end, we compiled site‐specific near‐surface air temperature data from 1982 to 1992 provided by the National Center for Environmental Prediction using the RNCEP package (Kemp et al. 2012). We calculated a daily temperature from the minimum and maximum out of the four 6‐h‐averages per day. Finally, the daily temperatures from 1982 to 1992 were averaged to a daily 11‐year mean temperature.

### Statistical analysis

We analysed the data in two steps: (1) the geographic model with wing and tarsus length related to longitude and latitude, and (2) the environment model with wing length related to migration distance (Dist), spring phenology measures (GU_time_, GU_speed_) and food availability (FA_first_, FA_high_). For both steps, we applied a hierarchical meta‐analysis model. The site‐ and sex‐specific averages of wing or tarsus length *y*_*i*_ were assumed to be normally distributed with a mean corresponding to the unknown (latent) site‐specific mean *θ*_*i*_ and a standard deviation corresponding to the standard error of the average wing or tarsus length se(*y*_*i*_). The *θ*_*i*_ were modeled as a normally distributed random variable with mean *μ*_*i*_ linearly dependent on the predictor variables. This part of the model is a normal multiple regression with the latent variable *θ*_*i*_ as outcome variable instead of direct observations. In this way, we account for variable precisions in our data set (measured as standard error).

$$y_{i}∼\text{Norm}\;\left(\theta_{i},\text{se}\left(y_{i}\right)\right),$$

$$\theta_{i}∼\text{Norm}\;\left(\mu_{i},\sigma \right),$$

μ=βX.

The predictor *β*X differed between the geographic and the environment model. For the geographic pattern in wing and tarsus length, we included the predictors latitude, longitude, sex, state of the bird (living or museum specimen) and interactions latitude × longitude, sex × latitude and sex × longitude. For the environmental pattern we considered Dist, GU_time_, GU_speed_, FA_first_, FA_high_ and sex, state of the bird and the two‐way interactions of Dist, GU_time_, GU_speed_, FA_first_, FA_high_ with sex, as predictors. In both models, we included a random site effect to account for potential measurement uncertainty per site.

All geographical and environmental data were *z*‐transformed. The categorical variable sex was used as a numeric variable, with males obtaining a value of zero and females of one. Individuals with nonidentified sex obtained a value of 0.32, that is the proportion of females in the data set with known sex, because we assumed similar sex ratios at all sites. We removed the variable “state” from the model if its effect was not significant (as assessed by the 95% credible interval) and not relevant (below the measurement accuracy i.e. <0.1 mm). In the second model (environmental pattern), only interactions with posterior probabilities of the hypothesis H: *β *> 0 lower than 0.3 or higher than 0.7 were retained. We used standard residual plots, that is the quantiles of the residuals (*y*_*i–*_ *μ*_*i*_) against the theoretical quantiles to assess their normal distribution. Additionally, the residuals were plotted against the fitted values and every predictor variable to control for nonlinear relationships and general independency. Lastly, we plotted the square‐roots of the absolute values of the residuals against the fitted values to check for homogeneity of variance.

All models were fitted in a Bayesian framework using Markov chain Monte Carlo simulation with WinBUGS in the R‐package R2WinBUGS run in R 3.0.3 (www.r-project.org). We used flat priors, that is Norm(0, 10,000), for the model coefficients and Gamma(0.01, 0.01) for the variance parameters. We simulated two Markov chains with 30,000 iterations each; the first 10,000 iterations were discarded as burn‐in. From the remaining values every 5th was retained to describe the posterior distributions of the model parameters. Whether the Markov chains converged was judged visually and by the R‐hat value (Brooks and Gelman 1998). Additionally, we used classical statistical tests and give frequentist *P*‐values if not stated otherwise.

## Results

### Geographic pattern in morphometry

**Figure 2:**
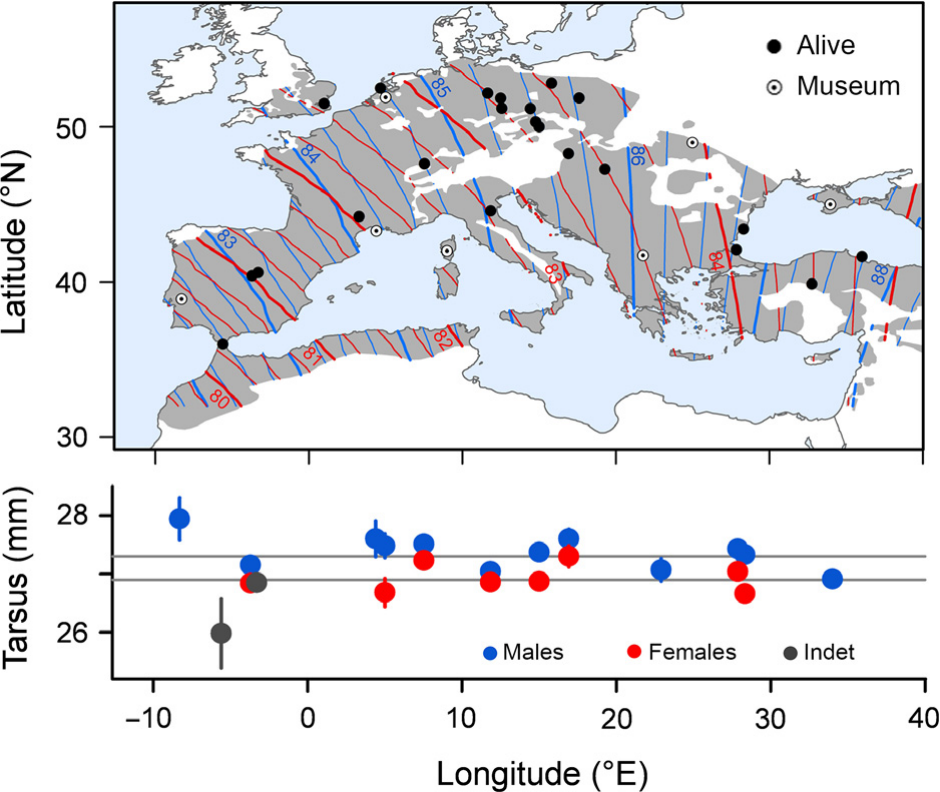
Geographically variable morphometry of common nightingales (*L. m. megarhynchos*) across the species breeding range. Upper panel: modeled sex specific variation in wing length (mm) with blue isolines for males and red isolines for females. The original sites of capturing are symbolized as black dots (data from living birds) and white dots (data from museum specimens). The distribution of the species (gray area) is based on IUCN (2012). Lower panel: the corresponding variation in average tarsus length (±SE). Gray dots are populations in which the sex of individuals was not determined, and gray lines indicate sex‐specific averages across the study populations.

Wing length varied between 83.1 and 89.2 mm for males and between 80.3 and 86.3 for females across the distribution range (Tables 1 and S1). We found a significant positive association between wing length and longitude with shorter wings in western populations and longer wings in eastern populations (Fig. 2), but we did not find a similar relation with latitude or any significant interactions of longitude, latitude and sex (Table 1). However, the longitude × latitude interaction was slightly negative (−0.16), resulting in left‐skewed wing‐length isolines when projected across the species range (Fig. 2). The fixed effects of the model explained on average 90% of the variance. The wing length data set was not biased by study years (regression of standardized deviations from expected wing length size for a given longitude against mean study year: *r* ^2^ = 0.05, *F*_1,24 _= 1.24, *P* = 0.28 for males, *r* ^2^ = 0.17, *F*_1,17_ = 3.26, *P* = 0.09 for females). Thus, we could exclude a directional bias caused by study years or by historically different measurement approaches for wing length.

**Table 1:** Summary statistics of the regression coefficients in the hierarchical model 1 (geographic model) for wing and tarsus length in common nightingales. For each trait and population sex is included as explanatory variable. Significant differences from zero are given in bold, Cr.I. is the credible interval. Data for longitude (Long) and latitude (Lat) were *z*‐transformed

	Wing length (*n* = 47)		Tarsus length (*n* = 22)	
	Mean	95% Cr.I.	Mean	95% Cr.I.
Intercepts: overall			27.3 ± 0.11	27.1/27.6
Intercept: status “alive”	85.3 ± 0.28	84.7/85.8	na	
Intercept: status “museum”	84.5 ± 0.50	83.5/85.5	na	
Sex (female)	−**2.35 **±** 0.14**	−**2.54**/−**1.98**	−**0.40 **±** 0.15**	−**0.70**/−**0.11**
Long	**1.10 **±** 0.34**	**0.44**/**1.76**	−0.12 ± 0.16	−0.42/0.18
Lat	0.16 ± 0.26	−0.34/0.66	0.07 ± 0.14	−0.20/0.33
Long × Lat	−0.16 ± 0.30	−0.75/0.43	−0.17 ± 0.15	−0.47/0.13
Sex × Long	−0.26 ± 0.18	−0.61/0.11	0.01 ± 0.15	−0.31/0.29
Sex × Lat	0.16 ± 0.16	−0.15/0.49	−0.04 ± 0.19	−0.41/0.33

In addition, tarsus length differed significantly between the sexes with the males’ tarsus exceeding the females’ tarsus by 0.4 ± 0.15 mm, but variation in tarsus length could not be related to longitude (Fig. 2), latitude or any interaction (Table 1). Mean *R* ^2^ goodness of fit for the tarsus model was 0.13.

### Geographic pattern of migration distances and environmental conditions

The migration distances of nightingale populations varied between 2500 and 4550 km, with shortest and longest distances in the western and northern‐central European populations respectively. However, migration distances did not correlate with longitude (*r* = 0.28, *P* = 0.10, *n* = 29, Fig. S1).

Spring phenology, that is the time of spring green up and its speed, were highly correlated with longitude (GU_time_: *r*_s_ = 0.68, *P* = 0.001, GU_speed_: *r* = 0.59, *P* =0.001, *n* = 29): the earliest and slowest green‐up occurred in Portugal and Spain, that is at the westernmost part of the distribution range (Fig. S1). The latest and fastest green‐ups were found in eastern Bulgaria and the Crimea Peninsula, that is at the eastern edge of distribution range (Fig. S1). The difference in GU_time_ between western and eastern breeding sites averaged 10–12 weeks; the speed of green‐up was about six times faster at the eastern compared to western breeding sites. Finally, both proxies for food availability did not relate with longitude (both FA_first_ and FA_high_: *P* = 0.31, *P* = 0.10, *n* = 29; Fig. S1).

### Wing morphometry, migration distances, and environmental conditions

**Figure 3:**
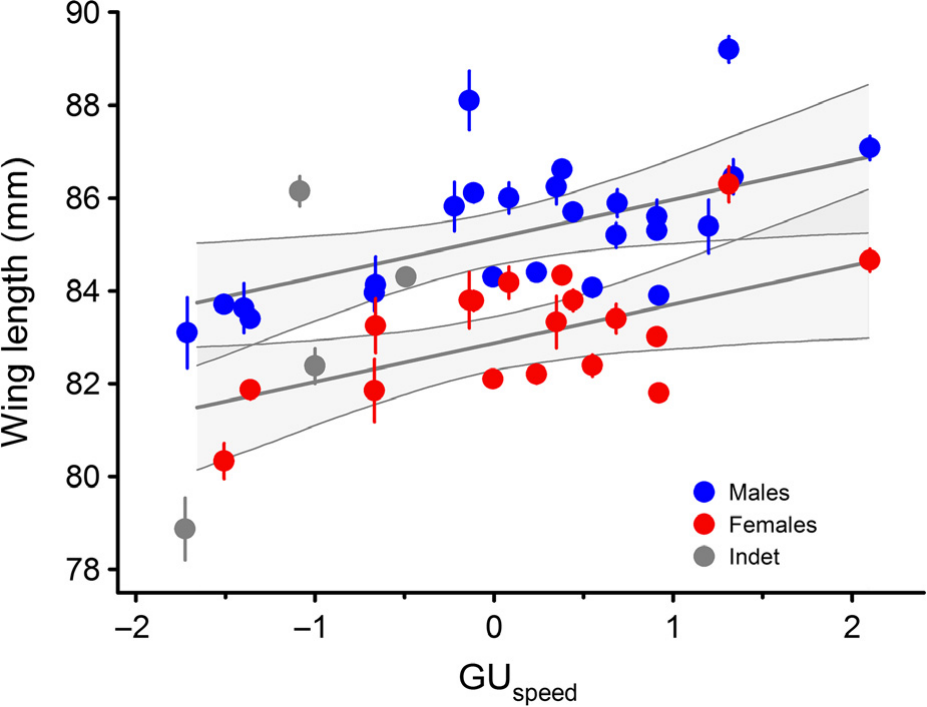
Mean wing length (mm, ±SE) of common nightingales from 28 breeding populations in relation to the speed of spring green‐up (GU_speed_) at their respective breeding site. Blue dots symbolize males, red dots females and gray dots birds from populations where sex was not determined. Lines give regression estimates ±95% credible intervals derived from the hierarchical model (see the “Statistical analysis” section in the “Materials and Methods” for details).

We failed at finding a significant relationship between the minimum migration distance and wing length across the range of the nightingale (Table 2). However, wing length and the speed of spring green‐up (GU_speed_) were positively related, that is birds from populations breeding in regions with a slow green‐up of vegetation in spring had significantly shorter wings than birds from areas with rapidly increasing plant productivity in spring (Fig. 3). The time of local green‐up was not related to wing length. Finally, we did not find a statistically significant relation between wing length and the time of first and peak food availability in spring (Table 2), but there was a positive interaction between the time of first food availability and sex (Table 2). The average goodness of fit of the environmental model was 49% (95% CI: 9–72%).

**Table 2:** Summary statistics of the regression coefficients in the hierarchical model 2 (environmental model) for wing length in common nightingales. Separate intercepts for the two states (alive and museum) were fitted. The environmental factors were minimum migration distance (Dist), spring phenology (time and speed of spring green‐up, GU_time_ and GU_speed_) and the first time and the time of high food availability (FA_first_ and FA_high_) at the respective breeding sites. Coefficients which differed significantly from zero (as assessed by the 95% Cr.I.) are given in bold, Cr.I. is the credible interval

Wing length	Mean	95% Cr.I.
Intercept ‐ status “alive”	85.1 ± 0.28	
Intercept ‐ status “museum”	85.0 ± 0.58	
Sex	−**2.26 **±** 0.13**	−**2.51**/−**2.02**
Dist	−0.05 ± 0.32	−0.65/0.63
GU_time_	0.33 ± 0.42	−0.48/1.17
GU_speed_	**0.84 **±** 0.37**	**0.11**/**1.54**
FA_first_	−0.53 ± 0.54	−0.89/0.58
FA_high_	0.89 ± 0.48	−0.07/1.80
FA_first_ × sex	0.31 ± 0.15	0.0/0.60

An extension of the environmental model using a quadratic effect, thus testing for a nonlinear relationship, did not substantially improve the results. The regression coefficients for GU_time_ remained non‐significant (mean: 0.33; 95%CI: −0.08, 0.76), and the GU_speed_ got marginally significant (mean: −0.88; 95%CI: −1.55, 0.25). However, this effect disappeared when excluding the three populations with unknown sex (mean: 0.07; 95%CI: −0.68, 0.8).

## Discussion

We verified for an obligatory long‐distance migrant that its wing length but not its structural body size increased longitudinally from the western to the eastern parts of the species range (Stresemann 1920; Eck 1975). More importantly, we extended the simple spatial concept by substituting the geographic scale with ecologically relevant parameters namely the speed of spring green‐up at breeding sites. Thus, the identification of potential drivers for morphological adaptation may allow for future predictions on phenotypic changes in populations which are subject to with differential variation in environmental conditions.

Variation in body size is often related to local environmental conditions, especially to temperature limitations in endothermic species (Bergmann's rule, Meiri and Dayan 2003) including body size and wing length variation in resident birds (e.g., Johnston and Selander 1972; Ashton 2002; Perktaş 2011). However, migratory birds can avoid unfavorable periods by seasonal movements, and thus aerodynamic attributes like flight costs may more importantly influence phenotypic variation (Leisler and Winkler 2003). In our study species, wing length, an aerodynamically relevant trait, varied along a longitudinal gradient, but structural body size as characterized by tarsus length did not.

Longer wings result in higher aspect ratios and thus in less energy required per flight distance at the same speed, or in higher flight speed while holding energy consumption constant (e.g., Norberg 1995). Both the energetic costs and the flight speed are crucial for the optimal timing of arrival at the breeding sites: lower energetic costs at the same speed decrease overall migration duration because stopover time for fuelling is reduced (Alerstam 2011; Nilsson et al. 2013). Long‐winged birds can achieve higher speeds compared to short‐winged counterparts with the same amount of fuel. As a consequence, flight speed and total migration speed can be adapted more flexibly. This is advantageous for individuals travelling along routes where conditions ahead are less predictable but arrival at final destination must be timed precisely. The phenomenon that longer‐winged individuals arrive earlier on the breeding grounds in at least some species (Stolt and Fransson 1995; Potti 1998; Cooper et al. 2011) may be explained by the ability of those individuals to migrate faster.

**Figure 4:**
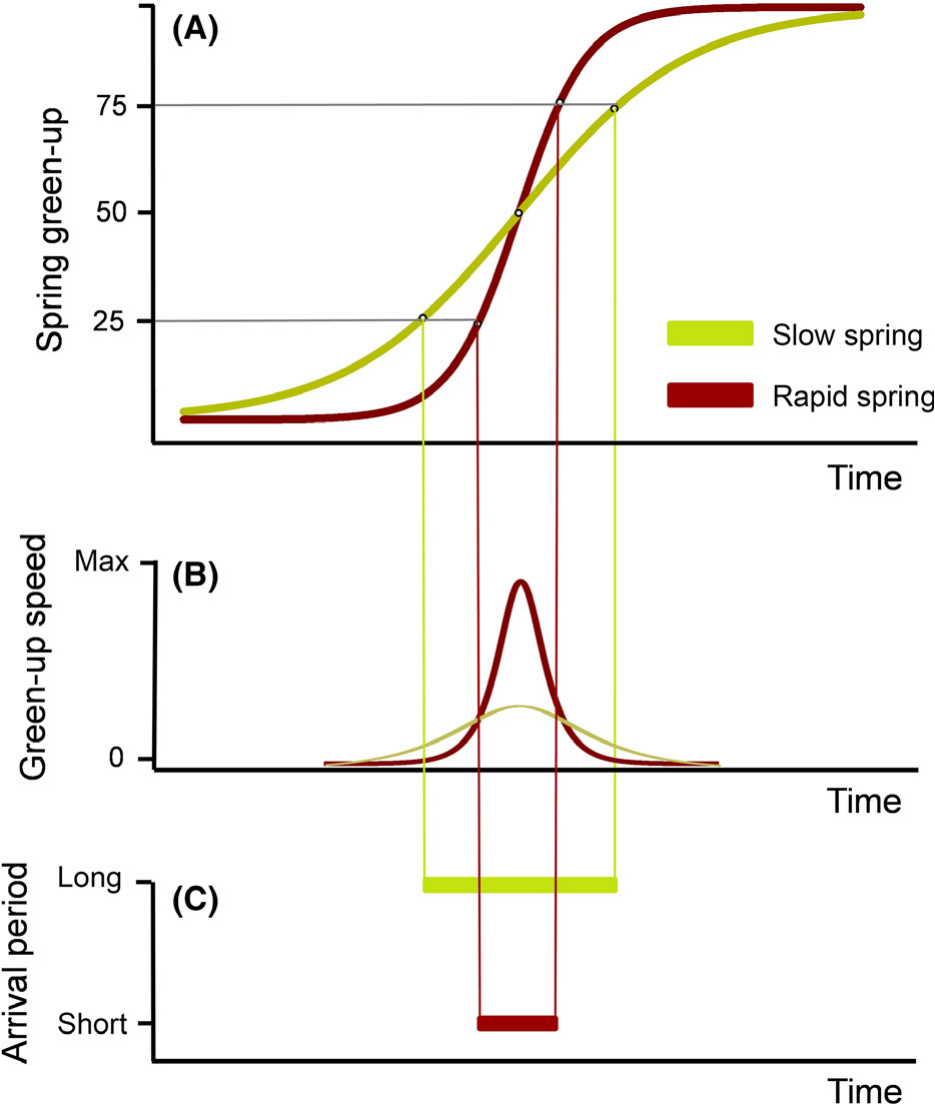
Concept of the relation between optimal arrival at a breeding site and (A) the local spring green‐up, with slow spring (green) and rapid spring (red). The onset of spring is defined as 50% of green‐up; the lines encompass the 25–75% quantiles around the onset of spring. (B) gives the corresponding green‐up speed derived from the logistic regression of spring green‐up over times. (C) visualizes the length of optimal arrival period within the quartile range which is considerably shorter at rapid spring green‐up sites (red) than at sites with slow green‐up (green).

Local conditions, for example at European breeding sites, may be hardly predictable from the sub‐Saharan nonbreeding sites (but see Saino and Ambrosini 2008 for air temperatures). Thus, birds can only obtain information about the progress of spring when they have crossed the Sahara desert (Balbontín et al. 2009). However, timely arrival at breeding sites is fundamental for reproduction, that is for finding high quality territories and to mate (Kokko 1999). The deviation from optimal arrival times, esp. late arrival, can drastically affect fitness (e.g., Cooper et al. 2011). Moreover, migrants are known to time their arrival in accordance with spring phenology and the peak in food availability (Drent 2006). Our results now add the speed of spring green‐up to the range of potential determinants for migratory timing. For a migratory bird breeding in habitats with rapidly increasing conditions in spring, that is high green‐up speed (Fig. 4A and B), the window for optimal arrival at the breeding site is much narrower than for individuals breeding at sites with slow green‐up (Fig. 4C). Spring green‐up in Europe typically vary considerably between years (Chmielewski and Rötzer 2002; Menzel et al. 2005). Under such variability longer wings should be beneficial for adjusting migration progression en route, that is speed‐up in advanced springs, to still arrive in the optimal period.

In Europe, especially at lower latitudes, regional climate becomes more continental from west to east with increasing distance to the Atlantic Ocean (Walter and Breckle 1994). Thus, the geographic longitude parallels the gradient from oceanic to continental climate on our study area, and this includes smaller differences between winter and summer air temperatures in the western compared to the eastern part, but also a more sudden temperature increase in spring, with concomitant patterns in vegetation and insect phenology. The seasonal increase of primary production based on ambient temperature and precipitation regimes can be nicely tracked using the composite NDVI. For the temporal pattern of food availability we used a single measure instead, for example the ambient air temperature because insect development in temperate regions is mainly driven by temperature (Jarošík et al. 2011). However, our proxies did not explain the variation in wing length across the continent and thus a multifactorial, composite measure as NDVI may better describe the temporal food availability in breeding habitats.

Many studies found positive intraspecific relationship of migration distance and wing length supporting the predictions of aerodynamic theory (i.e., Pérez‐Tris and Tellería 2001; Fiedler 2005; Milá et al. 2008; Förschler and Bairlein 2011). For instance, the wing length of blackcaps (*Sylvia atricapilla*) increased rapidly by about 8% from sedentary to migratory populations with an average migration distance of 2000 km, but it nearly levelled off when considering distances >2000 km (Pérez‐Tris and Tellería 2001). In our study, we found a 7% increase in wing length between exclusively long‐distance migrating populations. Thus, the population specific travel costs between nonbreeding and breeding sites are most likely not the prime factor for the development of longer wings towards the eastern part of the species range. Although we are not aware of any study relating green‐up speed with wing length in migratory birds, the relation between GU_speed_ and wing length might be more generally present. Our study species might be especially suitable for such a test, because its core distribution encompasses western, central and south‐eastern Europe without spreading towards northern parts of the continent and the migration distances of various populations range from 2500 to 4500 km. In species whose migration distances increase considerably towards the north‐eastern parts of Europe (caused by the uneven distribution of the land masses across longitudes), the effect of higher green‐up speeds on wing length adaptation might be concealed by the parallel increase in migration distance and thus may have remained undetected so far.

The interpretation of our results is based on two premises: the between‐observer variation of measurements does not affect the geographical pattern in morphology; and wing length is strongly affected by requirements for efficient migratory flights, whereas tarsus length represents structural body size. There are indirect hints that observer variation is not important in our study. If there would be such an error, we would expect a random noise of measurements instead of a cline from western to eastern populations. Additionally, an earlier study carried out on museum specimen (Eck 1975) found that individuals from eastern populations had on average longer wings than birds from western populations supporting the pattern we identified for the entire range of the nominate nightingale.

Body size, which is synonymous for structural size (Piersma and Davidson 1991), is best quantified using a combination of skeletal and/or external measurements (e.g., Freeman and Jackson 1990; Senar and Pascual 1997; Perktaş 2011). For practical and ethical reasons, field ornithologists prefer simple external measurements to quantify structural size. However, studies using principal component analysis of skeletal measurements found that tarsus length was well‐correlated with skeletal size, whereas wing length was a less appropriate measure (Rising and Somers 1989; Freeman and Jackson 1990; Senar and Pascual 1997; but see Gosler et al. 1998 for lean mass/size relations). Since we found no geographical difference in tarsus length, we conclude that structural body size does not vary across our studied populations and the differences in wing length indicate adjustments to regionally variable requirements for migratory flight performance.

Ecomorphologists have long agreed that variation in migration distance is a major factor promoting morphological differentiation among populations of migratory animals (e.g., Leisler and Winkler 2003). Our results may expand this paradigm by acknowledging the role of environmental heterogeneity at destination in fine‐tuning bird phenotypes.

## Data Accessibility

All data used in this manuscript are present in the manuscript and its supporting information.

## Conflict of Interest

None declared.

## Supporting information


**Table S1.** Morphometry of nightingales (*Luscinia m. megarynchos*) across its distribution range.
**Figure S1.** Longitudinal pattern of migration distance and four environmental factors at specific breeding sites of local populations used in the study.
