# Supplementary material for: Longer wings for faster springs – wing length relates to spring phenology in a long‐distance migrant across its range
Source: Ecol Evol. 2015 Dec 8;6(1):68–77. doi: 10.1002/ece3.1862 (PMC4716511; doi:10.1002/ece3.1862)
Supplement: Supplementary file 1 — Table S1. Morphometry of nightingales (Luscinia m. megarynchos) across its distribution range. Figure S1. Longitudinal pattern of migration distance and four environmental factors at specific breeding sites of local populations used in the study. [file ECE3-6-068-s001.docx]

Supporting Information, Appendix 1

Table S1: Morphometry of nightingales (*Luscinia m. megarynchos*) across its distribution range. Data are population means ± SD, n gives the number of individuals, the category ‘unisex’ refers to data where the sex had not be determined. Years give the time of study.

| Region | Longitude | Latitude | Wing length (mm) | | | | | | Tarsus length (mm) | | | | | | Years | Reference |
| --- | --- | --- | --- | --- | --- | --- | --- | --- | --- | --- | --- | --- | --- | --- | --- | --- |
|  | (°E) | (°N) | males | n | females | n | unisex | n | males | n | females | n | unisex | n |  |  |
| PT | -8.3 | 38.9 | 83.10±1.7 | 5 |  |  |  |  | 27.94±0.81 | 5 |  |  |  |  | 1938 | 1 |
| ES – south | -5.6 | 36.0 |  |  |  |  | 78.87±2.42 | 13 |  |  |  |  | 25.98±2.29 | 15 | 1995-1999 | 2 |
| ES – central | -3.7 | 40.4 | 83.40±2.26 | 235 | 81.87±2.14 | 113 |  |  | 27.15±0.67 | 233 | 26.84±0.79 | 112 |  |  | 2006-2009 | 3 |
| ES - central north | -3.3 | 40.6 |  |  |  |  | 82.38±2.34 | 38 |  |  |  |  | 26.85±0.76 | 34 | 1995-1999 | 2 |
| GB - southeast | 1.0 | 51 - 52 | 84.30±2.10 | 298 | 82.10±2.10 | 155 |  |  |  |  |  |  |  |  | 1900-2011 | 4 |
| FR - south 1 | 3.28 | 44.22 | 84.13±2.36 | 15 | 83.25±1.86 | 10 |  |  |  |  |  |  |  |  | 2000-2012 | 5 |
| FR - south 2 | 4.4 | 43.3 | 83.63±1.08 | 4 |  |  |  |  | 27.60±0.61 | 4 |  |  |  |  | 1953/1954 | 1 |
| NL – west | 4.67 | 52.5 |  |  |  |  | 84.30±1.90 | 173 |  |  |  |  |  |  | 1960-2006 | 6 |
| NL | 5.0 | 51.9 | 83.96±1.46 | 14 | 81.85±2.14 | 10 |  |  | 27.48±0.71 | 12 | 26.68±0.76 | 10 |  |  | 1842-1970 | 1 |
| FR - central east | 7.53 | 47.62 | 84.07±2.22 | 128 | 82.39±2.13 | 82 |  |  | 27.51±0.83 | 128 | 27.23±0.77 | 81 |  |  | 2009-2010 | 7 |
| FR – Corsica | 9.0 | 42.0 | 83.71±0.38 | 12 | 80.33±1.16 | 9 |  |  |  |  |  |  |  |  | 1907 | 8 |
| DE - east 1 | 11.65 | 52.17 | 84.40±2.60 | 336 | 82.20±2.50 | 164 |  |  |  |  |  |  |  |  | 1980-2004 | 9 |
| IT – north | 11.85 | 44.58 | 86.11±2.08 | 111 | 83.79±1.72 | 67 |  |  | 27.04±0.96 | 111 | 26.86±0.78 | 67 |  |  | 2009-2010 | 7 |
| DE - east 2 | 12.5 | 51.85 | 85.70±1.50 | 250 | 83.80±1.82 | 66 |  |  |  |  |  |  |  |  | 1999-2004 | 10 |
| DE - east 3 | 12.57 | 51.2 | 85.20±1.90 | 50 | 83.40±1.80 | 32 |  |  |  |  |  |  |  |  | 1981-2004 | 9 |
| DE - east 4 | 14.43 | 51.18 | 83.90±1.81 | 101 | 81.80±1.61 | 90 |  |  |  |  |  |  |  |  | 1984-1990 | 11 |
| CZ - north west 1 | 14.78 | 50.32 | 85.60±1.99 | 30 |  |  |  |  |  |  |  |  |  |  | 2007 | 12 |
| CZ - north west 2 | 15.0 | 50.0 | 85.30±1.89 | 277 | 83.01±1.66 | 124 |  |  | 27.37±0.77 | 277 | 26.87±0.80 | 124 |  |  | 2006-2012 | 13 |
| PL - north west | 15.82 | 52.82 | 85.82±1.75 | 11 |  |  |  |  |  |  |  |  |  |  | 2007 | 12 |
| AT – east | 16.92 | 48.27 | 89.20±1.90 | 45 | 86.30±2.00 | 27 |  |  | 27.60±0.80 | 24 | 27.30±0.70 | 15 |  |  | 1975-1978 | 14 |
| PL – west | 17.61 | 51.86 | 85.89±1.74 | 35 |  |  |  |  |  |  |  |  |  |  | 2007 | 12 |
| HU – central | 19.25 | 47.25 | 86.62±2.39 | 505 | 84.34±1.99 | 246 |  |  |  |  |  |  |  |  | 1983-2012 | 15 |
| MK- central | 21.77 | 41.72 | 86.24±1.84 | 25 | 83.33±1.37 | 6 |  |  |  |  |  |  |  |  | 1918 | 16 |
| UA – west | 22.9 | 48.4 | 86.46±1.38 | 14 |  |  |  |  | 27.07±0.72 | 14 |  |  |  |  | 1904-1974 | 17 |
| BG - south east | 27.86 | 42.08 | 87.08±2.58 | 98 | 84.66±1.80 | 55 |  |  | 27.43±0.82 | 98 | 27.04±0.81 | 55 |  |  | 2009-2010 | 7 |
| BG - north east | 28.33 | 43.42 | 86.00±2.27 | 47 | 84.18±1.53 | 20 |  |  | 27.33±0.65 | 47 | 26.66±0.60 | 20 |  |  | 2009-2010 | 7 |
| TR – central | 32.78 | 39.87 | 88.10±1.68 | 7 | 83.80±1.91 | 10 |  |  |  |  |  |  |  |  | 2003-2005 | 18 |
| Crimea | 34.0 | 45.0 | 85.39±2.70 | 22 |  |  |  |  | 26.91±0.53 | 22 |  |  |  |  | 1947-1972 | 17 |
| TR - north central | 35.95 | 41.73 |  |  |  |  | 86.15±2.01 | 39 |  |  |  |  |  |  | 2002-2012 | 19 |

**References**

1: C.S. Roselaar, unpubl. data, Naturalis Biodiversity Centre, Leiden, The Netherlands.

2: Tellería, J. L., Pérez-Tris, J. and Carbonell, R. 2001. Seasonal changes in abundance and flight-related morphology reveal different migration patterns in Iberian forest passerines. - Ardeola 48: 27-46.

3: J. Pérez-Tris, unpubl. data, Department of Zoology and Physical Anthropology, Complutense University of Madrid, Spain.

4: British Trust of Ornithology: <http://blx1.bto.org/birdfacts/results/bob11040.htm#biometrics>

(accessed 07.2012)

5: O. Duriez, unpubl. data, CEFE/CNRS, Montpellier, France.

6: Levering, H. P. A. and Keijl, G. O. 2008. Vinkenbaan Castricum 1960-2006 - een halve eeuw vogels ringen. VRS Castricum, Castricum.

7: S. Hahn, unpubl. data, Swiss Ornithological Institute, Sempach, Switzerland.

8 + 16: Stresemann, E. 1920 Avifauna Macedonica: die ornithologischen Ergebnisse der Forschungsreisen, unternommen nach Mazedonien durch Prof. Dr. Doflein und Prof. L. Müller-Mainz in den Jahren 1917 und 1918. von Dultz, München. (incl. references therein).

9: Dorsch, H. 2010. Zur Biometrie von Kleinvögeln. - Mitteilung Vereins Sächsischer Ornithologen 10: 1-275.

10: Schönfeld, M. 2004. Zu Vorkommen, Bestandsentwicklung und Biometrie der Nachtigall (*Luscinia megarhynchos* C.L.Brehm 1831) in Gehölzresten des Elbtales um Wittenberg im Vergleich mit anderen Gebieten. Naturwissenschaftliche Beiträge Museum Dessau 16: 5-27.

11: Deunert, J. 1993. Zur Flügellänge Oberlausitzer Nachtigallen (*Luscinia megarhynchos*) (Aves, Passeriformes: Turdidae). Zoologische Abhandlungen Staatliches Museum für Tierkunde Dresden 47: 205-211.

12: Reifova, R., Reif, J., Antczak, M. and Nachman, M.W. 2011. Ecological character displacement in the face of gene flow: Evidence from two species of nightingales. BMC Evolutionary Biology 11: 138.

13: P. Kverek, unpubl. data, Kněžmost, Czech Republik.

14: A. Grüll, pers. com. & in Glutz von Blotzheim, U.N. (ed) (1988) Handbuch der Vögel Mitteleuropas Vol. 11/I. Aula-Verlag Wiesbaden.

15: T. Csörgö, unpubl. data, Ocsa Ringing Station, Hungary.

17: Loskot, V.M. 1981. [On the subspecies of the nightingale (*Luscinia megarhynchos* Brehm)]. Trudy Zoologitscheskovo Instituta Akademji Nauk SSSR, 102, 62–71. (re-measured by A. Poluda, University of Kiev, in 2012)

18: Özbahar, I. 2005. Breeding biology, population size and spatial distribution of a Common Nightingale (*Luscinia megarhynchos* Brehm, 1831) population at Yalincak (Ankara). Middle East Technical University Ankara. MSc thesis.

19: A. Gurzoy, unpubl. data, Cernek Ringing Station, Turkey.

Figure S1

Figure S1: Longitudinal pattern of migration distance and four environmental factors at specific breeding sites of local populations used in the study. All data were z-standardized.
